# Convolutional Neural Networks and Geometric Moments to Identify the Bilateral Symmetric Midplane in Facial Skeletons from CT Scans

**DOI:** 10.3390/biology10030182

**Published:** 2021-03-02

**Authors:** Rodrigo Dalvit Carvalho da Silva, Thomas Richard Jenkyn, Victor Alexander Carranza

**Affiliations:** 1 Craniofacial Injury and Concussion Research Laboratory, Western University, London, ON N6A 3K7, Canada; tjenkyn@uwo.ca (T.R.J.); vcarranz@uwo.ca (V.A.C.); 2 School of Biomedical Engineering, Faculty of Engineering, Western University, London, ON N6A 3K7, Canada; 3 Department of Mechanical and Materials Engineering, Western University, London, ON N6A 3K7, Canada; 4 School of Kinesiology, Faculty of Health Sciences, Western University, London, ON N6A 3K7, Canada; 5 Wolf Orthopaedic Biomechanics Laboratory, Fowler Kennedy Sport Medicine Clinic, London, ON N6A 3K7, Canada; 6 School of Biomedical Engineering, Faculty of Engineering, Collaborative Specialization in Musculoskeletal Health Research, and Bone and Joint Institute, Western University, London, ON N6A 3K7, Canada

**Keywords:** craniofacial skeleton, cephalometric analysis, convolutional neural network, geometric moments

## Abstract

**Simple Summary:**

The bilateral symmetry midplane of the facial skeleton plays a critical role in reconstructive craniofacial surgery. By accurately locating the midplane, surgeons can use the undeformed side of the face as a template for the malformed side. However, the location of the midline is still a subjective procedure, despite its importance. This study aimed to present a 3D technique for automatically calculating the craniofacial symmetry midline of the facial skeleton from CT scans using deep learning techniques. A total of 195 skull images were evaluated and were found to be reliable and provided good accuracy in symmetric images.

**Abstract:**

In reconstructive craniofacial surgery, the bilateral symmetry of the midplane of the facial skeleton plays an important role in surgical planning. Surgeons can take advantage of the intact side of the face as a template for the malformed side by accurately locating the midplane to assist in the preparation of the surgical procedure. However, despite its importance, the location of the midline is still a subjective procedure. The aim of this study was to present a 3D technique using a convolutional neural network and geometric moments to automatically calculate the craniofacial midline symmetry of the facial skeleton from CT scans. To perform this task, a total of 195 skull images were assessed to validate the proposed technique. In the symmetry planes, the technique was found to be reliable and provided good accuracy. However, further investigations to improve the results of asymmetric images may be carried out.

## 1. Introduction

Craniomaxillofacial reconstructive surgery is a complex and difficult multidisciplinary technique due to the intricate anatomy of the skull. The aim of craniomaxillofacial reconstruction surgery is to preserve the patient’s appearance, strengthen facial functions, and regain the bilateral symmetry of the craniofacial skeleton. However, craniomaxillofacial reconstructive surgery may lead to complications such as bone disorders, congenital deformities, trauma, pathologies, genetic abnormalities, and cancers. A precise recognition of the bilateral symmetry facial midplane is an imperative step for pre-surgical planning and implant design techniques. For facial restoration, this midplane plays a major role when one side of the image is replicated and used as a guide to recreate the deformed or injured side. The authors of [1] have established a widely agreed approach for defining the midline of the craniofacial skeleton. To date, however, the most popular two-dimensional image application method, or the midsagittal plane (MSP) for a three-dimensional object, is the method introduced by [2].

There are a few approaches that aim to simplify the task of locating the midline plane of the facial skeleton. One technique proposed by [3] describes a semiautomatic system that, in conjunction with surface models reconstructed from computed tomography images (CT), uses principal component analysis (PCA) and the iterative closest point (ICP) alignment method. The first step is to determine the direction of the mirror plane correctly. This was achieved by using PCA to match the replicated mesh and the initial mesh roughly. Then, the ICP algorithm was described by a refined registration. The downside of this approach was the dependency on the central point of the image for the approximation of the symmetrical plane (obtained using the average of the vertices of the facial mesh). If the central point was in the wrong position due to any external factors (such as imperfect symmetry), this approach would lead to a symmetrical plane in the wrong direction and position. In addition, this algorithm is not able to adjust and learn from previous images to improve its performance, limiting its capabilities. 

Alternatively, [4] determines the midline symmetry plane by using boney landmarks to create a midline representing facial symmetry. For a stack of horizontal lines crossing bilaterally through the facial skeleton containing boney landmarks, this approach essentially measures the midline symmetry plane as a perpendicular midpoint. This approach involves the manual collection of a variety of cephalometric boney landmarks in the dataset by either specifically locating the landmarks on the plane (which requires great attention by an expert user) or by using the midline as a reference and locating the landmarks at equal distances from the midline. However, manual skeletal landmark selection is ineffective, time-consuming, and reliant on an expert operator, resulting in errors in the measurement of the symmetry plane [5] outlines an ICP-based process for automated symmetry plane detection of 3D asymmetrically scanned human faces that uses particle swarm optimization. This approach starts with a discrete 3D model. The symmetry plane is tested by a tentative first attempt using a PCA algorithm. The model is then refined iteratively by a Levenberg–Marquardt algorithm before its final prediction is obtained. This revised version enhances the shortcomings of [6], but the current implementation also struggles to integrate self-learning to maximize the result of the model and misses the ability to learn from previous versions.

By minimizing the error-index of the symmetry plane, an automated method based on an iterative process was recently proposed by [7]. To automatically correct the initial symmetry plane, with a significant contribution to the use of the rotation matrix derived from the registration process, this method performs analytical data analysis in 3D point sets derived from CT images. First, the plane was divided into two groups by the initial symmetry plane estimated by the PCA and the collection of skull points. Then, to match two point-sets, the ICP registration method was used.

Most recently, ref. [8] introduced a novel automatic concept for determining the bilateral symmetry midline of the facial skeleton based on invariant moments. This technique creates a dataset from images aligned using cephalometric landmarks. The images are then rotated from 14° to 15° with a resolution of 0.5°. Then, after comparing different feature extractors, pseudo-zernike moments were selected for having the best accuracy using the k-nearest neighbors classifier. Finally, after detecting the rotation degree of the image, the midpoint is calculated using geometric moments. However, this model still has some limitations. For instance, this method uses 2D images with an image resolution of 128 × 128 which becomes difficult in real applications on different image modalities such as computed tomography and magnetic resonance imaging (MRI). Additionally, this technique was not tested on non-symmetrical skull images which may affect its results.

**Figure 1:**
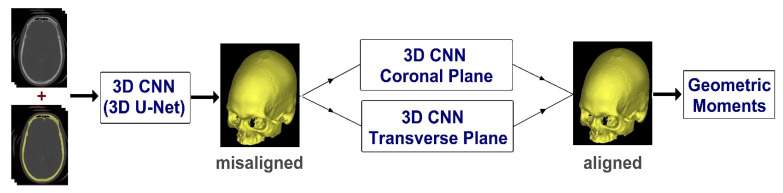
Overview of the proposed method. CNN: convolutional neural network.

Thus, this study aims to present a 3D technique for automatically calculating the craniofacial symmetry midplane from CT scans using convolutional neural network (CNN) and geometric moments. Figure 1 shows the overview of the proposed method. First, using 3D U-net, the skull is removed from CT images to create a dataset. Then, based on the cephalometric landmarks, the CT image is aligned in the coronal and transverse planes. The image is then duplicated and two datasets of 441 images, per image, of 0.5° resolution is created from −5° to 5°. These sets of images are presented to a 3D rotate invariant CNN. After CNN determines the rotation degree of these images in the coronal and transverse planes, the skull midpoints are calculated using 3D geometric moments. Finally, by joining the midpoints and grades described by the CNN, the midplanes can be constructed.

## 2. Materials and Methods

### 2.1. Data Processing

**Figure 2:**
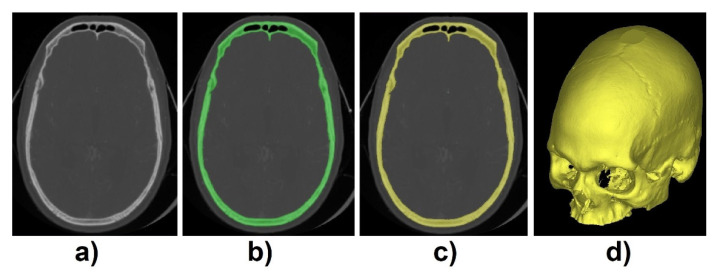
(**a**) Computed tomography (CT) scan, (**b**) thresholding applied, (**c**) region growing, and (**d**) 3D mesh model.

The dataset used to validate the proposed method was acquired from the qure.ai CQ500 dataset [9]. From this dataset, 195 images with 512 × 512 and varied depths were selected for training, validation, and test purposes. To create the ground truth labels, CT images were imported into Mimics Medical Imaging Software (Materialise, Leuven, Belgium). First, individual thresholding with manual corrections was applied for each of the 3D volumetric CT images. Then, region growing was applied to create the 3D model mesh. This process allowed for the creation of the standard tessellation language (STL) file format which was converted into a matrix using voxelization method [10] so we can easily process the file in MATLAB R2019b software (Mathworks, USA) (Figure 2).

### 2.2. CNN Architecture and Implementation Details

#### 2.2.1. CNN Framework for Biomedical Image Segmentation

The framework chosen in this paper for biomedical image segmentation was the U-Net [11]. U-Net has been used in a number of biomedical image segmentation applications such as kidney segmentation [12], prostate and prostate zones segmentation [13], brain tumor segmentation [14], brain segmentation [15], and so forth. Its name emerged from the idea of a U-shape architecture where in the first step, downsampling path, the spatial information is reduced while feature information is increased. In the next step, upsampling path, contracting path concatenate high-resolution features with spatial information and features. The result is a CNN that can work with few training samples and has the possibility to apply large images. We adopted a 3D U-net modified version of the code [16] initially implemented for brain tumor segmentation in MRI. The parameters adopted in this work are presented in Table 1. These parameters were chosen to avoid computational crash and error, while obtaining a good accuracy for the training set explored in this work.

#### 2.2.2. CNN Framework for Rotation Invariant

By nature, CNNs are not rotation invariant, however, with a combination of convolutional, max pooling, average pooling, relu, and fully connected layers, the CNN framework can be transformed into rotation invariant. A number of papers have exploited the rotation invariant [17,18,19], however, the adopted framework presented in Table 2 worked very well in the dataset proposed using the Adam optimizer and a mini-batch size of 128.

Both CNN models were performed on an Intel i7-9700 (3.00 GHz) computer with 64 gigabyte (GB) of ram memory, and two 8GB Video RAM graphics processing units (GPUs) from NVIDIA (one RTX 2070 SUPER and one RTX 2080). The source code was implemented and tested in MATLAB R2019b.

### 2.3. Model Performance Evaluation and Statistical Analysis

For biomedical segmentation evaluation, the Dice Similarity Coefficient (DSC) [20] is the most popular metric to evaluate segmentation models. The DSC is a statistical method to gauge the similarity between two sample sets. In biomedical segmentation, the DSC measures the overlap between the ground truth and the predicted segmentation where 0 represents no overlap and 1 indicates complete overlap. Equation (1) defines the DSC, where the area of overlap is divided by the total pixels combined (TP—true positives, FP—false positives, and FN—false negatives).

(1) $$\mathrm{DSC}\;=\;\frac{\mathrm{Area}\;\mathrm{of}\;\mathrm{Overlap}}{\mathrm{Total}\;\mathrm{Pixels}\;\mathrm{Combined}}=\;\frac{2\mathrm{TP}}{2\mathrm{TP}\;+\;\mathrm{FP}\;+\;\mathrm{FN}}.$$

Symmetric Volume Difference (SVD) [21,22] is the corresponding error metric.


SVD = 1 − DSC.
(2)

Hausdorff Distance (HD) is a size-based method that describes the maximum distances between the boundaries of the segmented regions and the ground truth. This metric can be defined as:

(3) $$\mathrm{HD}\;=\;\max\left(h\left(S,\;\mathrm{GT}\right),\;h\left(\mathrm{GT},\;S\right)\right),$$

where $h\left(S,\mathrm{GT}\right)\;={\;\max}_{a\in S}\min_{b\in \mathrm{GT}}||a-b||$ [23].

To evaluate the performances of the CNN framework for rotation invariant, we implemented the following measures: average difference (AD), image quality index (IQI), Laplacian mean square error (LMSE), maximum difference (MD), mean-squared error (MSE), normalized absolute error (NAE), normalized cross-correlation (NK), structural content (SC), and structural similarity index (SSIM). To evaluate the classification, a sensitivity analysis was performed by using positive predictive value (PPV) and negative predictive value (NPV) defined as:

(4) $$\mathrm{Sensitivity}=\frac{\mathrm{TP}}{\mathrm{TP}+\mathrm{FN}},$$

(5) $$\mathrm{Specificity}=\frac{\mathrm{TN}}{\mathrm{TN}+\mathrm{FP}},$$

(6) $$\mathrm{PPV}=\frac{\mathrm{TP}}{\mathrm{TP}+\mathrm{FP}},$$

(7) $$\mathrm{NPV}=\frac{\mathrm{TN}}{\mathrm{TN}+\mathrm{FN}}.$$

### 2.4. 3D Geometric Moments

To avoid manual intervention, 3D geometric moments is applied for the automatic extraction of the central point. Three-dimensional $\left(p+q+r\right)\mathrm{th}$ order moments of a digitally sampled 3D image that has the gray function $f\left(x,y,z\right)$ [24] is given as:

(8) $$M_{\mathrm{pqr}}=\underset{x}{\sum }\underset{y}{\sum }\underset{z}{\sum }x^{p}y^{q}z^{r}f\left(x,\;y,\;z\right),$$

where p,q,r=0, 1, 2, 3, …. As described in [25], the mass and area of the zeroth order moment, $M_{000}$, of a digital image is defined as:

(9) $$M_{000}=\underset{x}{\sum }\underset{y}{\sum }\underset{z}{\sum }f\left(x,\;y,\;z\right),$$

The center of mass of the image $f\left(x,y,z\right)$ is represented by the two first moments:

(10) $$M_{100}=\underset{x}{\sum }\underset{y}{\sum }\underset{z}{\sum }\mathrm{xf}\left(x,\;y,\;z\right),$$

(11) $$M_{010}=\underset{x}{\sum }\underset{y}{\sum }\underset{z}{\sum }\mathrm{yf}\left(x,\;y,\;z\right),$$

(12) $$M_{001}=\underset{x}{\sum }\underset{y}{\sum }\underset{z}{\sum }\mathrm{zf}\left(x,\;y,\;z\right),$$

Thus, the centroid of an image can be calculated by:

(13) $$\;\bar{X}=\frac{M_{100}}{M_{000}},\;\bar{Y}=\frac{M_{010}}{M_{000}},\;\mathrm{and}\;\bar{Z}=\frac{M_{001}}{M_{000}}.$$

As best practice, the center of mass was chosen to represent the position of an image in the field of view. The centroid of the image $f\left(x,y,z\right)$, given by Equation (13), can be used to describe the position of the image in space by using the point as a reference point

## 3. Results and Discussion

### 3.1. Skull Segmentation

The CT volumetric dataset and 3D mesh models were presented to the 3D U-Net with the parameters described by Table 1. From the 195 images, 190 were used for training and 5 for validation/testing. Table 3 shows the DSCs, SVDs, and HDs, in terms of mean ± standard deviation (SD) after being trained and tested 10 times, acquired from the testing set. When using two GPUs as specified, the CNN took 57 min in 15 epochs to converge.

**Figure 3:**
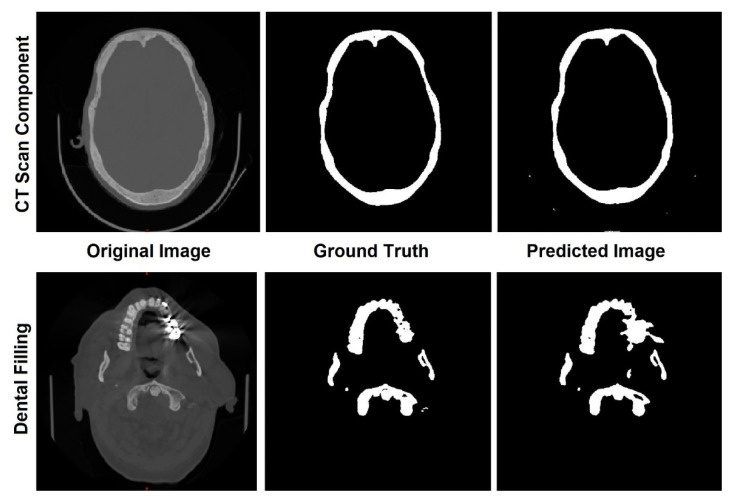
Errors in the predicted images caused by machine components and dental fillings.

These results are close to those DSCs reported by [26] (mean DSC of 0.92), and slightly lower than the results reported by [27] (mean DSC of 0.98). Regarding HD, its discrepant values may be directly related to segmentation errors due to bright artifacts found in the original image, which may be caused by dental filling and components of the CT scan machine, as shown in Figure 3. However, any necessary modification was performed by manual corrections after the segmentation. These predicted labels play an important role in the coronal and transverse alignment.

### 3.2. Transverse and Coronal Angles

**Figure 4:**
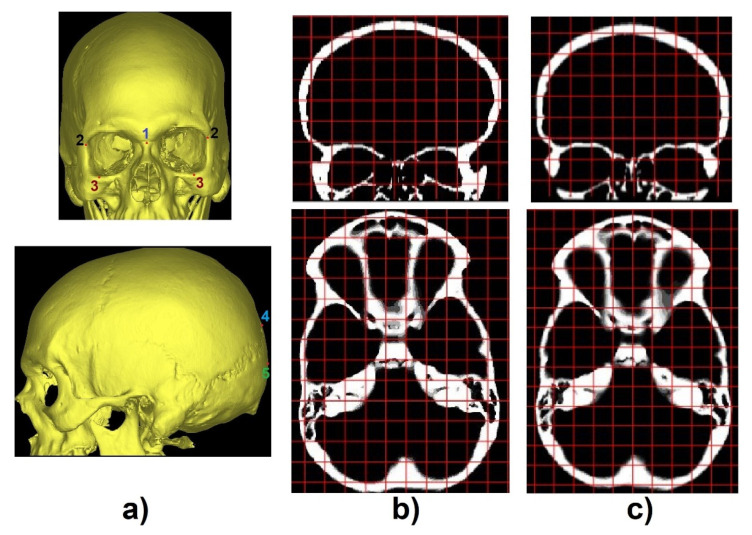
(**a**) Identification of cephalometric landmarks in coronal (top) and sagittal plane (bottom), (**b**) CT slices misaligned, and (**c**) CT slices after the alignment procedure (coronal plane-top and transverse plane-bottom).

To identify the transverse and coronal angles through the 3D CNN, 101 volumetric images, from the 195 segmented images, were selected to create a database. The first step was to identify cephalometric landmarks to help align the predicted labels Figure 3a. In the coronal plane, we selected the 1-crista galli, 2-frontozygomatic suture, and 3-orbitale while in the sagittal plane, we selected 4-lambda and 5-opistocranion. To identify these points and make the necessary alignment, two grids were generated as a reference in the transverse and coronal planes. A number of slices were verified, and the necessary adjustments were performed (Figure 4b,c).

**Figure 5:**
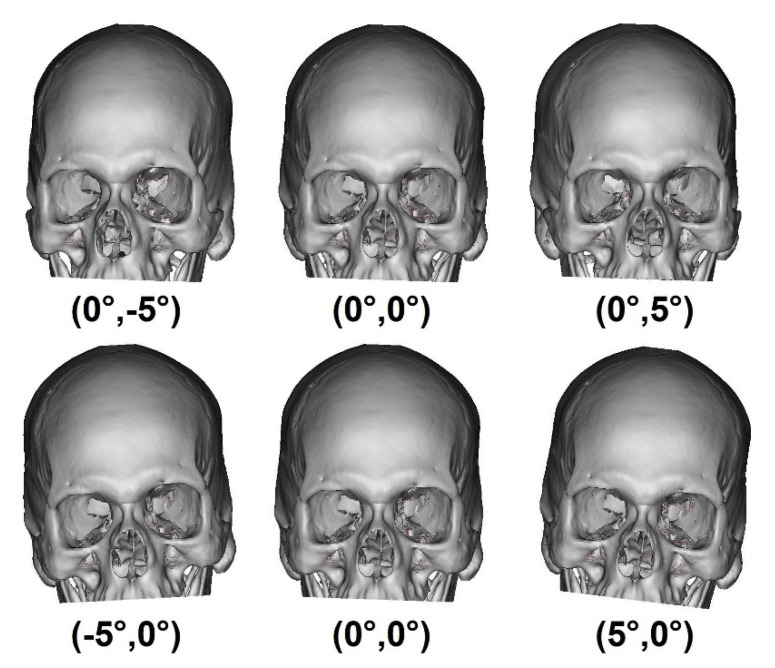
Examples of images rotated in the coronal and transverse planes where parentheses represents (coronal, sagittal), respectively.

After the alignment, for each of the 101 images, a set of 441 images with inclination angles from −5° to 5°, with 0.5° increments, along the coronal and transverse planes was created. In total, 44541 images were created and were divided into 21 labels. These labels represented the 0.5° of variation in the coronal and transverse planes from −5° to 5°, as shown in Figure 5.

**Figure 6:**
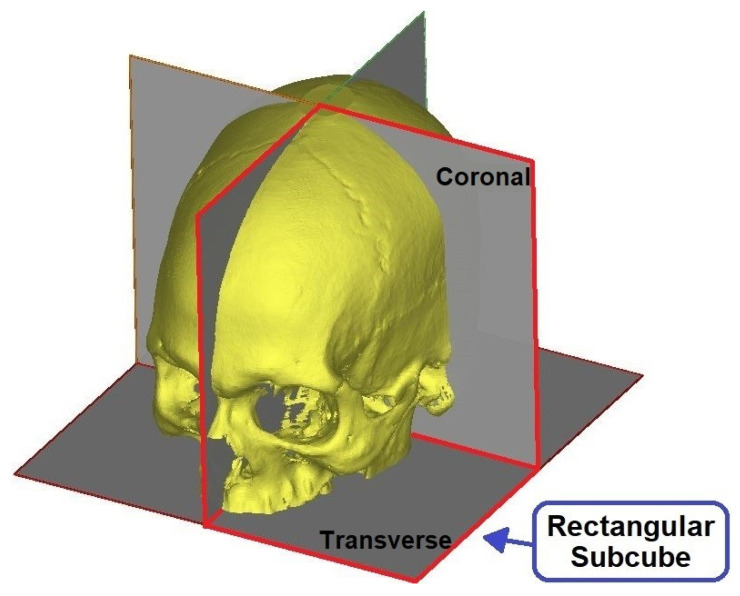
Volumetric image divided into rectangular subcubes and the selected one-quarter subcube.

**Figure 7:**
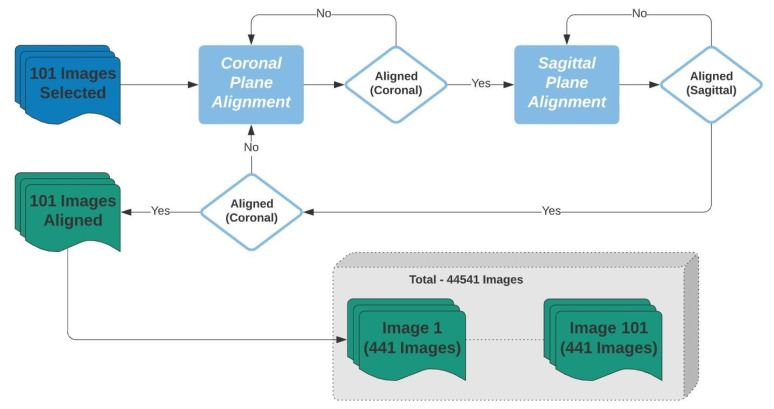
Flowchart representing the process of transversal and coronal alignment and database creation.

To reduce the computational and processing time, these images were reduced to 128 × 128 using the nearest neighbor interpolation method. Additionally, the volumetric image was divided into four rectangular sub-cubes and only one-quarter of the whole image space was used to predict the angles as shown in Figure 6. As this step aims to identify the coronal and transverse angles in symmetrical skulls, these steps do not affect the output image. Figure 7 summarizes this process.

In this phase, we used the 3D rotation invariant CNN introduced in Section 2.2.2 and detailed in Table 2. To optimize the CNN convergence, computational time, and accuracy, two identical datasets were created using the 44,541 images and represented by 21 labels. Thus, 21 labels represent the rotation in the transverse plane and 21 labels in the coronal 201 plane. 90% of the dataset was used for training, and 10% for validation/testing. Training and testing were performed 10 times. Table 4 shows the analytical performance for these two. It took 30 min in five epochs for the transverse and 70 min in 12 epochs for the coronal CNN to converge using two GPUs.

As seen in Table 4, we can see that CNN performed well and can represent a rotation invariant image descriptor for these scenarios. In fact, the accuracy reached ≈99% with simple hyperparameter, which allows the construction of a simple 3D CNN. After the discovery of the transverse and coronal deviation angles, the original image was then rotated accordingly with these two found angles.

### 3.3. Geometric Moments Image Center

**Figure 8:**
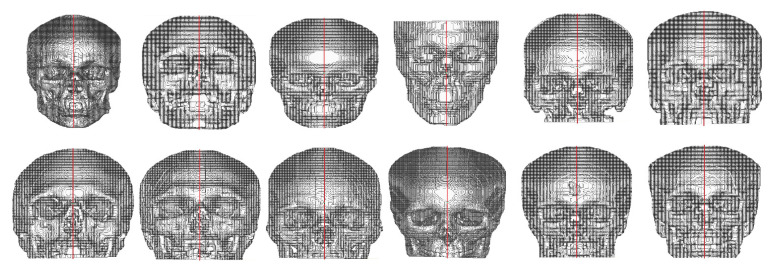
12 samples with their respective cross-sectional plane created using geometric moments.

**Figure 9:**
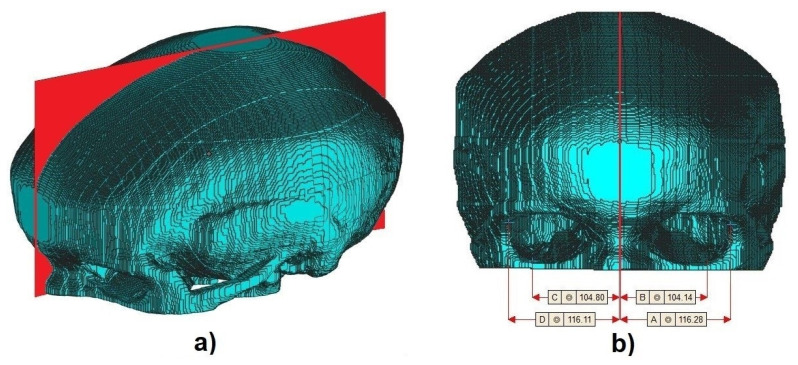
(**a**) Perspective and (**b**) front view.

Finally, to calculate the center of the volumetric images, Equations (8) to (13) were used. As there are no patterns to validate the accuracy of the center-point, visual evaluation was used and compared with cephalometric landmarks. Figure 8 and Figure 9 show the cross-sectional plane created from the geometric moments. Figure 9a presents the perspective view and Figure 9b shows the front view of one aligned sample with measured dimensions of frontozygomatic suture and orbitale in the sagittal plane displayed in Materialise MiniMagic software. We used [28] to convert from voxel into STL file format.

### 3.4. Deformed Skull Test

**Figure 10:**
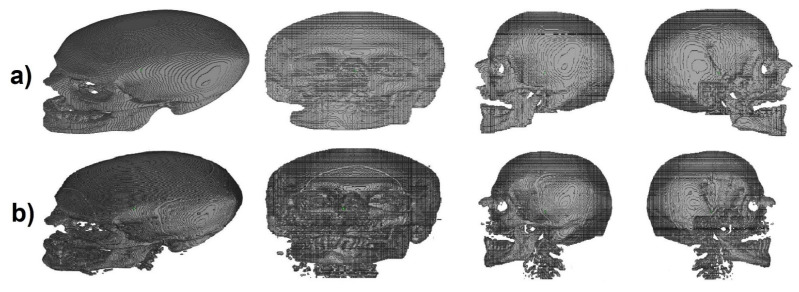
(**a**) Ground truth and (**b**) predicted.

To validate this method in deformed images, eight defected CT images were used from two different datasets [29,30,31,32] found in the cancer imaging archive data collections (TCIA) [33]. In the first step, STL files were generated by the 3D U-Net using the same parameters as the Table 1. DSC, SVD, and HD results are presented in Table 5 and a sample is shown in Figure 10.

The CQ500 database does not contain deformed images which may have caused the discrepancy in the DSC results. Unfortunately, there are no databases of deformed skulls for analysis. Furthermore, the 195 images used for training were not enough to improve the 3D U-Net prediction. Reference [27] reported a 6% mean improvement compared to [26] and associated the improvement with the size of the image dataset for training purposes. Moreover, in the sampled image (Figure 10), part of the vertebral column and small segmented parts that do not belong to the skull were segmented by mistake, which generated the worst DSC value and high HD values. This is likely associated with the C1 vertebrae labeled during the creation of the ground truth and small bright artifacts on the outside of the skull in the CT scans. Even though it seems like an error, this can be disregarded since Figure 10 shows that the ground truth and predicted label to be very close.

**Figure 11:**
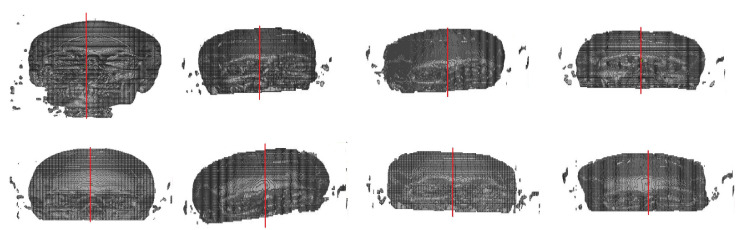
Results of the implemented method in deformed/asymmetric images.

Finally, using the eight models predicted, transverse and coronal angles were calculated, and the center of the image was acquired using 3D geometric moments. Figure 11 shows the results for the deformed images.

The proposed method shows good results in obtaining the bilateral symmetric midplane of regular/symmetric images. However, for deformed images, it failed to identify the rotation for some images along with the image center. These errors are likely due to some factors:

- the small database size, which is already reported in [27];
- to the best of our knowledge, there are no deformed CT database available which restricts the possibility to train the system with deformed images;
- during the ground truth segmentation process and voxelization, a few regions of interest (ROIs) may have not been incorporated in the 3D model. The first may be caused by the manual selection of the ROI, performed by an expert, which leads to the CNN generating the defects. Secondly, a quantity of information from the skull voxel may be lost due to the smoothing of the edges and noise residuals removal performed in the segmentation process;
- regarding the center of the 3D images, as reported by [8], when the image suffers from a lack of symmetry, non-uniform brightness, deformation, interference, or incompleteness, the calculation of the image center using geometric moments becomes complex and finds some restrictions as this technique is a quantitative measure of an image’s function or structure.

It is possible to state that the proposed method obtained good results from symmetric CT images datasets. However, for deformed images, an improvement is necessary to achieve better results. For this purpose, an increase in the CT database size may be performed as well as the inclusion of non-uniform and deformed CT images which may also lead to an improvement in the detection of the transverse and coronal angles.

Finally, a modification in the method to identify the center of the image may be carried out by creating a 3D U-net to segment the nasal bone instead of the presented geometric moments technique. This method will allow for the definition of the center of the image using the center of the nasal bone.

## 4. Conclusions

This study aimed to introduce a 3D automatic technique for determining the craniofacial symmetry midplane from CT scans using the convolutional neural network and gemetric moments. A total of 195 symmetric CT images were used to evaluate this method using the CQ500 database while eight asymmetric CT images from TCIA database were used to evaluate the performance in asymmetric images. For symmetric images, this method obtained results close to 99%. However, for asymmetric images, the method needs further development to improve its results. CNNs offer an effective alternative to the pseudo-zernike moments method and conventional landmark-based symmetry scores that depend on the general positions of cephalometric landmarks. CNNs are also an alternative to PCA-ICP techniques, which depend on the manual selection of the central point which cannot be improved. With the proposed technique, the central point could be found as the centroid of an image, and then the symmetrical midplane can be constructed for symmetric images. In this study, we have shown the proposed technique to be reliable and to provide the midplane symmetry plane with great accuracy in symmetric images. This method can be used as a tool to aid surgeons in reconstructive craniofacial surgeries.

## Figures and Tables

**Table 1:** Skull CT segmentation implementation details.

Parameter	Value
Optimizer	Adam
Encoder Depth	4
Filter Size	5
Number of First Encoder Filter	6
Patch Per Image	4
Min Batch Size	128
Initial Learning Rate	10^−2^

**Table 2:** Rotation-invariant CNN framework adopted.

	Layers	Size	Number Filter	Stride
	3D Conv	1 × 1	3	1
1	BN + Relu	-	-	-
	3D Max pooling	5 × 5	-	2
	3D Conv	5 × 5	8	1
2	BN + Relu	-	-	-
	3D Max pooling	5 × 5	-	2
	3D Conv	7 × 7	16	1
3	BN + Relu	-	-	-
	3D Max pooling	3 × 3	-	2
	3D Conv	5 × 5	32	1
4	BN + Relu	-	-	-
	3D Max pooling	2 × 2	-	2
	3D Conv	5 × 5	64	1
5	BN + Relu	-	-	-
	3D Average pooling	2 × 2	-	2
6	3D Conv	1 × 1	128	1
	BN + Relu	-	-	-
7	FC (25 neurons)	-	-	-
	Relu	-	-	-
8	FC (50 neurons)	-	-	-
	Relu	-	-	-
9	FC (labels neurons)	-	-	-
	Softmax	-	-	-

**Table 3:** Skull and Background DSCs, SVDs, and HDs Values of 5 samples and their mean. DSC: Dice Similarity Coefficient; SVD: Symmetric Volume Difference; HD: Hausdorff Distance.

DSC-Skull	DSC-Background	SVD-Skull	HD-Skull
0.8993 ± 0.004	0.9927 ± 0.0003	0.1007 ± 0.004	67.7 ± 09.20
0.9093 ± 0.008	0.9948 ± 0.0005	0.0907 ± 0.008	27.81 ± 31.19
0.9150 ± 0.008	0.9941 ± 0.0006	0.0850 ± 0.008	38.78 ± 20.26
0.9349 ± 0.008	0.9958 ± 0.0005	0.0651 ± 0.008	49.92 ± 37.69
0.9362 ± 0.006	0.9953 ± 0.0004	0.8844 ± 0.006	39.99 ± 44.40
0.9189 ± 0.016	0.9945 ± 0.0012	0.0811 ± 0.016	44.73 ± 14.79

**Table 4:** Statistical analysis of the coronal and transverse CNNs. PPV: positive predictive value; NPV: negative predictive value; AD: average difference; IQI: image quality index; LMSE: Laplacian mean square error; MD: maximum difference; MSE: mean-squared error; NAE: normalized absolute error; NK: normalized cross-correlation; SC: structural content; SSIM: structural similarity index.

Index	Ideal Value	Coronal CNN Value	Transverse CNN Value
Accuracy	1	0.9909 ± 0.0038	0.9947 ± 0.0034
Sensitivity	1	0.9811 ± 0.0170	0.9969 ± 0.0054
Specificity	1	0.9982 ± 0.0012	0.9994 ± 0.0005
PPV	1	0.9646 ± 0.0236	0.9892 ± 0.0106
NPV	1	0.991 ± 0.0009	0.9998 ± 0.0003
AD	0	0.0124 ± 0.0085	0.0004 ± 0.0095
IQI	1	0.9979 ± 0.0014	0.9994 ± 0.0004
LMSE	0	0.9926 ± 0.6717	0.9571 ± 0.0814
MD	0	11.667 ± 1.1547	9.3333 ± 5.6862
MSE	0	0.2710 ± 0.1883	0.0723 ± 0.0561
NAE	0	0.0030 ± 0.0018	0.0012 ± 0.0007
NK	1	0.9985 ± 0.0010	0.9998 ± 0.0004
SC	1	0.9982 ± 0.0008	1.0000 ± 0.0008
SSIM	1	0.9285 ± 0.0313	0.9523 ± 0.0290

**Table 5:** Skull and background DSCs, SVDs, and HDs values of the 8 defected skulls and their mean.

DSC-Skull	DSC-Background	SVD-Skull	HD-Skull
0.8206 ± 0.080	0.9902 ± 0.0003	0.1794 ± 0.080	48.56 ± 03.69
0.8114 ± 0.005	0.9831 ± 0.0011	0.1886 ± 0.005	49.71 ± 06.21
0.8294 ± 0.012	0.9875 ± 0.0007	0.1706 ± 0.012	47.57 ± 08.61
0.8626 ± 0.016	0.9890 ± 0.0008	0.1374 ± 0.016	46.60 ± 07.76
0.8974 ± 0.011	0.9946 ± 0.0004	0.1026 ± 0.011	49.20 ± 10.86
0.8302 ± 0.011	0.9817 ± 0.0010	0.1698 ± 0.011	56.17 ± 07.55
0.7888 ± 0.010	0.9898 ± 0.0004	0.2112 ± 0.010	50.81 ± 09.57
0.8361 ± 0.014	0.9891 ± 0.0012	0.1639 ± 0.014	34.27 ± 14.06
0.8346 *±* 0.033	0.9881 *±* 0.0041	0.1654 *±* 0.033	47.86 *±* 06.21

## Data Availability

The datasets presented in this study are openly available at: CQ500- http://headctstudy.qure.ai/dataset (accessed on 1 June 2020), and TCIA- https://www.cancerimagingarchive.net/ (accessed on 1 June 2020).

## Abbreviations

The following abbreviations are used in this manuscript:

AD	average difference
CNN	convolutional neural network
CT	computerized tomography
DSC	dice similarity coefficient
FN	false negative
FP	false positive
GB	gigabyte
GPUs	graphics processing units
HD	hausdorff distance
ICP	iterative closest point
IQI	image quality index
LMSE	laplacian mean square error
MD	maximum difference
MRI	magnetic resonance imaging
MSE	mean-square error
MSP	midsagittal plane
NAE	normalized absolute error
NK	normalized cross-correlation
NPV	negative predictive value
PCA	principal component analysis
PPV	positive predictive value
ROIs	regions of interest
SC	structural content
SD	standard deviation
SSIM	structural similarity index
STL	standard tessellation language
SVD	symmetric volume difference
TP	true positive
